# Layout optimization of irregular storage areas under class storage strategy based on clustering and multi-bin size packing problem

**DOI:** 10.1371/journal.pone.0307218

**Published:** 2024-08-30

**Authors:** Wenbin Zhang, Yuehua Jin, Ronghua Zhang, Yiming Wang

**Affiliations:** 1 School of Mathematical Sciences, Jiangsu Second Normal University, Nanjing, China; 2 Jiangsu Anfang Electric Power Technology Co., Ltd., Taizhou, China; Cyprus International University Faculty of Engineering: Uluslararasi Kibris Universitesi Muhendislik Fakultesi, TÜRKIYE

## Abstract

This paper proposes an optimization scheme for the layout of irregular warehouse spaces based on a class-based storage strategy. Firstly, we transform the irregular warehouse space into several regular rectangular areas. Next, through the class-based storage strategy, we develop an algorithm that converts the non-linear clustering problem of homogeneous shelves into a linear selection problem of different sized regular shelf areas. Finally, a comprehensive shelving clustering algorithm and packing problem with different box sizes selection were constructed, and empirical analysis was conducted based on actual data from Xiangtai Warehouse of State Grid Corporation of China. The results show that the new model not only effectively solves the irregular warehouse layout optimization problem under the class storage strategy but also reduces the complexity of the model and shortens the solution time. It is a universally applicable method with significant value for generalization.

## 1. Introduction

Warehouse operations are essential for ensuring the safety of the electrical grid. The construction and maintenance of the grid require a vast quantity and variety of materials, with a provincial-level power company’s bidding equipment quantity reaching several hundred thousand units per year [1]. A key aspect of warehouse operation is layout optimization. Efficient warehouse layouts can reduce material handling costs and improve space utilization [2]. Therefore, layout optimization has always been a hot topic in warehouse operations.

Based on different rules for item distribution, warehouse layouts can be classified into five different storage allocation strategies. Dedicated Storage Strategy: This involves sorting products based on demand rules and ranking positions according to the distance from input/output points, subsequently fixing the position of high turnover rate products at optimal locations [3]. However, the dedicated storage strategy cannot effectively use space because empty positions cannot be used for other products when product stocks are depleted. Shared Storage Strategy: A method to overcome this issue is using a shared storage strategy, whereby any product can be allocated an empty position. For example, a random strategy assigns pallets to any available position with equal probability [4]. While this random allocation leads to high utilization of storage space, it often results in longer travel distances. Class-Based Storage Strategy: To balance warehouse utilization and transportation distances, a class-based allocation strategy has been proposed [5], wherein products are classified into categories and assigned a fixed area in the warehouse; within the class, storage is random. Turnover-Based Storage Strategy: Additionally, strategies based on turnover have been studied [6]. Closest Open Position Storage Strategy: Furthermore, strategies concerning the nearest open location are also considered [7]. Currently, in warehouse management, class storage strategy is the most popular strategy.

Implementing a class-based storage strategy within a warehouse layout involves three key steps: (i) classification of materials, (ii) determining the size of each class’s area, and (iii) positioning each area within the layout [8]. There are primarily two approaches to warehouse layout using a class-based storage strategy. The most popular involves grouping products into classes based on order frequency, known as ABC storage [8]. Another approach is based on Systematic Layout Planning (SLP) which considers the interrelationships between work units [9]. To overcome the subjectivity and manual complexity of the SLP method, scholars have enhanced the intelligence of warehouse layout planning by integrating it with heuristic algorithms [10].

Despite the extensive research on class-based storage strategies for warehouse layouts, electrical material warehouses have unique characteristics and requirements. For instance, the classification of electrical materials is based on the type of shelving such as flat storage areas, automated racks, pallet zones, and outdoor storage yards. Additionally, due to requirements for grouping similar items together, clustering issues of similar shelving types must be considered. Furthermore, it is necessary to develop a model that optimizes the shape, size, and position of shelving areas to enhance the utilization of irregular warehouse spaces in practical scenarios.

Consequently, This paper proposes an optimization model that combines clustering and variable-size two-dimensional packing problem to solve the layout optimization problem of power supply warehouse based on class storage strategy [11]. This new method addresses gaps in the literature in the following ways: (1) It proposes an algorithm that converts the nonlinear clustering problem of homogeneous shelves into a linear selection problem of regular shelf areas of different shapes and sizes.(2) By converting irregular warehouse areas into multiple regular areas, integrating clustering and variable-size two-dimensional packing problem, a new model for layout optimization based on class storage strategy in irregular warehouse areas is proposed.

**Fig 1:**
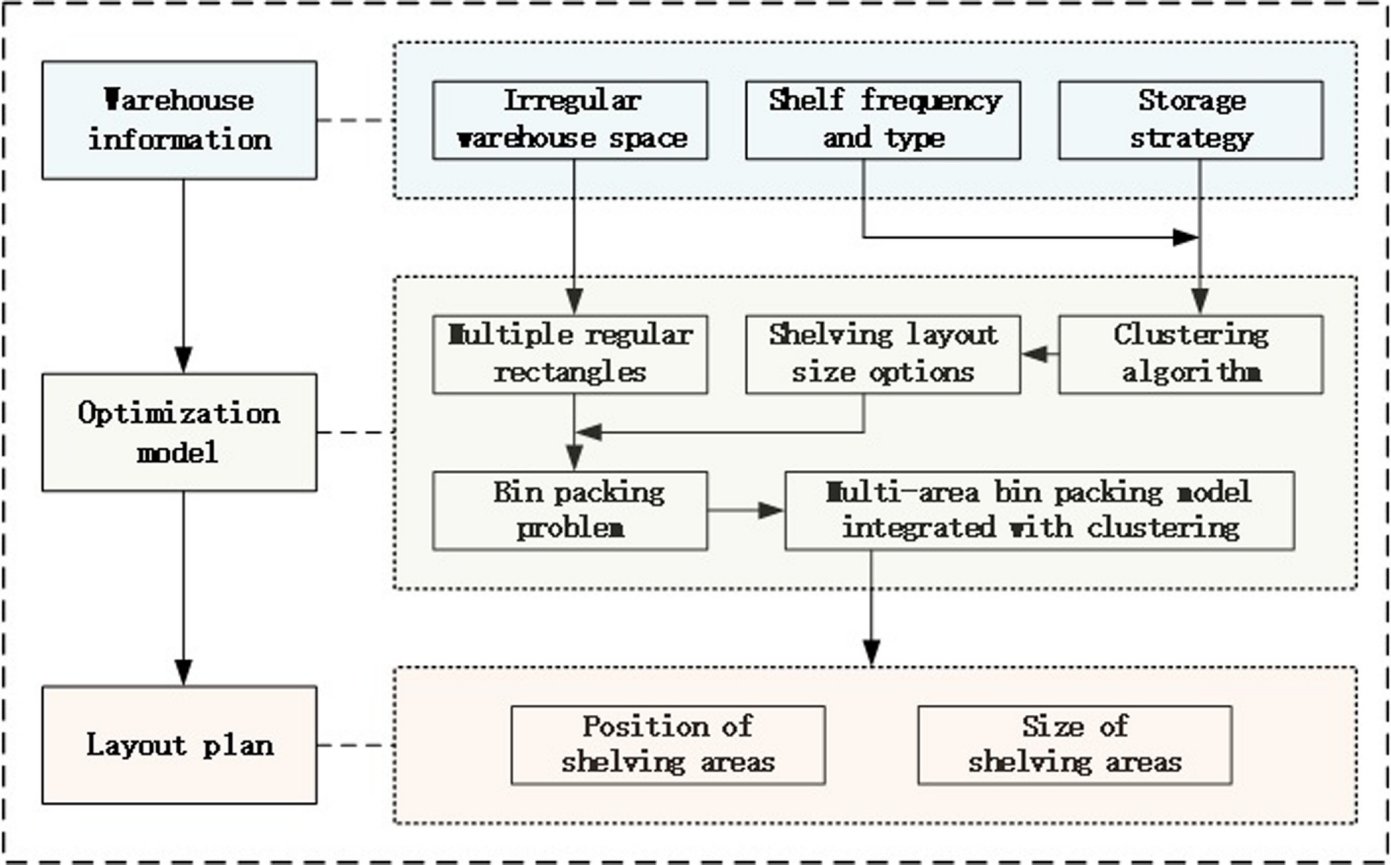
The flowchart of warehouse layout plan.

The flowchart of this paper is illustrated as shown in the figure below (Fig 1). The structure of this paper is as follows: Section 2 develops the layout optimization model; Section 3 provides empirical analysis; Section 4 offers a summary.

## 2. Model and methods

### 2.1. Symbols and Notations

The symbols and notations used in the algorithms and models are organized as Table 1:

**Table 1:** Symbols and notations.

Notations	
SAH	Algorithm Abbreviations of shelves arranged horizontally
AAS	Algorithm Abbreviations of arbitrary arrangement of shelves
**Parameters**	
*f*_*i*_	The frequency of the i-th shelf
*l*_*i*_	The length of the i-th shelf
*L*	The length of the entire warehouse space
*L*_0_	The maximum value of {*L*_*t*_}
*L*_*t*_	The length of the t-th rectangular region
*n*_1_	The frequency of corridors within the designated area
*p*_1_	The width of the walkway within the designated area
*p*_2_	The spacing between each row of shelves
*p*_3_	Gap width between shelves
*w*_*i*_	The width of the i-th shelf
*W*	The width of the entire warehouse space
*W*_0_	The maximum value of {*W*_*t*_}
*W*_*t*_	The width of the t-th rectangular region
*X*_*t*_	The x-axis of the bottom left corner of the t-th rectangular region
*Y*_*t*_	The y-axis of the bottom left corner of the t-th rectangular region
**Sets**	
*B*_1_	The set of all small rectangular sizes when arranging shelves horizontally
*B*_2_	The set of all small rectangular sizes when the shelves are horizontally arranged after 90 degree rotation
*B*_3_	The set of all small rectangular sizes when arranging shelves vertically
*B*_4_	The set of all small rectangular dimensions when the shelves are longitudinally arranged after 90 degree rotation
*B*	The union of set *B*_*i*_, *i* = 1,2,3,4.
*C*	The set of rectangles obtained by rotating the small rectangles in set *B* by 90 degrees
*BC*	Union of set *B* and set *C*
Variables	
*a*_*iji′j′*_	The 0–1 variable that determines the spatial left and right positional relationship between the j-th layout of the i-th type shelf and the *j*′-th layout of the *i*′-th type shelf
*b*_*iji′j′*_	The 0–1 variable that determines the spatial up and down positional relationship between the j-th layout of the i-th type shelf and the *j*′-th layout of the *i*′-th type shelf
*b*_*x*_	The length of small rectangles in the BRA algorithm
*b*_*y*_	The width of small rectangles in the BRA algorithm
*c*_*ijt*_	A 0–1 variable that determines whether the j-th layout method of the i-th shelf is placed in the t-th rule area
*e*1_*ij*	A 0–1 variable that determines whether the i-th row and the j-th row in the discrimination set *B* are identical
*e*2_*ij*	A 0–1 variable that determines whether the i-th row in set *B* is the same as the j-th row in set *C*
*floor*()	Function for rounding down (rounding off decimal parts)
*ceil*()	The function of rounding up (converting decimal parts to 1 and adding them to integers)
*mod*(*m*,*n*)	The integer part of the numerical value m divided by the numerical value n
*L*	A very large constant, this article takes 10000
${\left\{m_{ij}^{k}\right\}}_{k=1}^{2}$	A 0–1 variable indicating whether the j-th layout configuration of the i-th class of shelves is rotated by 90 degrees, with the sum of the two variables equaling 1.
*x*_*ij*_	The x-coordinate of the bottom-left corner of the j-th layout configuration of the i-th class of shelves in the warehouse.
*y*_*ij*_	The y-coordinate of the bottom-left corner of the j-th layout configuration of the i-th class of shelves in the warehouse.

### 2.2. Model preparation

#### 2.2.1. Transformation of irregular storage areas

Irregular storage areas not only increase the complexity of the optimization model but also the difficulty and time required for solving it. To address this, this article will transform the irregular storage areas into multiple regular rectangular regions: ${\left\{\left(L_{t},W_{t}\right)\right\}}_{t=1}^{T}$, where *L*_*t*_ represents the length of the t-th rectangular area, and *W*_*t*_ represents its width.

#### 2.2.2. Clustering algorithm for homogeneous shelves

Based on the need for concentrated placement of similar types of shelves, in this section, we will develop a clustering algorithm for shelves. Considering the integration of the shelving clustering algorithm with the two-dimensional bin packing optimization model, we opt for an arrangement approach, thereby converting the nonlinear clustering problem of homogeneous shelves into a linear problem of selecting regular shelving areas of different shapes and sizes.

Let *l*_*i*_,*w*_*i*_ and *f*_*i*_ represent the length, width, and frequency of the i-th type of shelf, respectively; *p*_1_ and *n*_1_ represent the width and frequency of the aisles, respectively; *p*_2_ represents the spacing between rows of shelves; *p*_3_ represents the gap between shelves. The algorithm for calculating the rectangular dimensions of shelves arranged horizontally (SAH) is as follows:

**Funfition** $SAH\left(l_{i},w_{i},f_{i},L_{0},W_{0},p_{1},p_{2},p_{3},n_{1}\right)$
 $I_{max}=min\left(floor\left(\left(L_{0}-n_{1}*p_{1}\right)/\left(l_{i}+p_{3}\right)\right),f_{i}\right)$// Determining the maximum number of shelves in SAH
 **for** *i* (from 1 to *I*_*max*_) // The number of iterations does not exceed *I*_*max*_
 $b_{x}=i\cdot \left(l_{i}+p_{3}\right)+n_{1}\cdot p_{1}$; // Calculation of the length of a rectangle
 $remainder=W_{0}-\left(w_{i}+p_{2}\right)-\left(2w_{i}+p_{2}\right)\cdot floor\left(\left(W_{0}-\left(w_{i}+p_{2}\right)\right)/\left(2w_{i}+p_{2}\right)\right)$// Intermediate variable
 **if** (*remainder* < *w*_*i*_) // For the scenario of having two rows of shelves together
  $b_{y}=\left(w_{i}+p_{2}\right)+\left(2w_{i}+p_{2}\right)\cdot floor\left(\left(ceil\left(f_{i}/i\right)-1\right)/2\right)$; //Calculation of the width of a rectangle
  **else if**  // Finally, for the situation of a single row of shelves
  $b_{y}=\left(w_{i}+p_{2}\right)+\left(2w_{i}+p_{2}\right)\cdot floor\left(\left(ceil\left(f_{i}/i\right)-1\right)/2\right)+mod\left(ceil\left(f_{i}/i\right)-1,2\right)\cdot w_{i}$// Width of the rectangle
 **end if**
 **end for**
 return *B*_*i*_ = {(*b*_*x*_,*b*_*y*_)}
**End**

Based on SAH algorithm, we have the following algorithm for arbitrary arrangement of shelves (AAS):

**Function** AAs(*l*_*i*_,*w*_*i*_,*f*_*i*_,*L*_0_,*W*_0_,*p*_1_,*p*_2_,*p*_3_,*n*_1_)
$B_{1}=SAH\left(l_{i},w_{i},f_{i},L_{0},W_{0},p_{1},p_{2},p_{3},n_{1}\right)$; // Horizontal arrangement
$B_{2}=SAH\left(w_{i},l_{i},f_{i},L_{0},W_{0},p_{1},p_{2},p_{3},n_{1}\right)$; // Horizontal arrangement allowing a 90-degree rotation
$B_{3}=SAH\left(l_{i},w_{i},f_{i},W_{0},L_{0},p_{1},p_{2},p_{3},n_{1}\right)$; // Vertical arrangement
$B_{4}=SAH\left(w_{i},l_{i},f_{i},W_{0},L_{0},p_{1},p_{2},p_{3},n_{1}\right)$; // Vertical arrangement allowing a 90- degree rotation
*B* = [*B*_1_,*B*_2_,*B*_3_,*B*_4_]; // Merge the above four sets into one large set
*C* = [*B*(2,:),*B*(1,:)]; // Swap the length and width values of rectangles in the set *B*
*BC* = [*B*,*C*]\; // Combine set *B* with set *C* to form a new set
**for** *i* (from 1 to *size*(*BC*,1)) // Iterations number does not exceed the number of rows in the set *BC*
 **for** *j* (from *i*+1 to *size*(*BC*,1))
 e1_*ij* = *isequal*(B(I,:),B(j,:)); // Determines whether the i-th row and the j-th row are identical
 **if** e1_*ij* = 1
  *index* = [*index*,*j*];// Record the serial number
 **end if**
  e2_*ij* = *isequal*(*B*(*i*,:),*C*(*j*,:)); // Determines whether the i-th row in set
   *B* and the j-th row in set *C* are identical
 **if** e2_*ij* = 1
  *index* = [*index*,*j*];// Record the serial number
  **end if**
  **end for** *j*
 **end for** *i*
 *BC*(:,*index*) = []; // Remove duplicate rows
 *B*_*i*__*all* = *BC*; // Record all combinations that allow a 90-degree rotation
**End**

#### 2.2.3. Layout optimization model

In this section, we will develop a layout optimization model for an electrical material warehouse based on the class storage strategy, utilizing the results of the aforementioned shelving clustering and irregular warehouse areas together with the two-dimensional bin packing problem [12].


$$min\;\sum_{t}W_{t}\cdot L_{t}-\sum_{i=1}^{N}\sum_{j=1}^{M_{i}}\sum_{t=1}^{T}c_{ijt}\cdot w_{i}\cdot l_{i}$$ (1.1)

$$s.t.\sum_{i=1}^{N}\sum_{j=1}^{M_{i}}{\text{c}}_{ijt}\geq 1,\forall t\left(t=1,\cdots ,T\right)$$ (1.2)

$$\sum_{j=1}^{M_{i}}\sum_{t=1}^{T}{\text{c}}_{ijt}=1,\forall i\left(i=1,\cdots ,N\right)$$ (1.3)

$$m_{ij}^{1}+m_{ij}^{2}=1,\forall i,\forall j$$ (1.4)

$$\left(x_{ij}-\sum_{t=1}^{T}c_{ijt}{\text{X}}_{t}\right)\leq \sum_{t=1}^{T}c_{ijt}L_{t}-m_{ij}^{1}\cdot l_{ij}-m_{ij}^{2}\cdot w_{ij},\forall i,\forall j;$$ (1.5)

$$x_{ij}\geq \sum_{t=1}^{T}c_{ijt}{\text{X}}_{t},\forall i,\forall j;$$ (1.6)

$$\left(y_{ij}-\sum_{t=1}^{T}c_{ijt}{\text{Y}}_{t}\right)\leq \sum_{t=1}^{T}c_{ijt}W_{t}-m_{ij}^{1}\cdot w_{ij}-m_{ij}^{2}\cdot l_{ij},\forall i,\forall j;$$ (1.7)

$$y_{ij}\geq \sum_{t=1}^{T}c_{ijt}{\text{Y}}_{t},\forall i,\forall j;$$ (1.8)

$$x_{ij}-x_{i^{\prime }j^{\prime }}+L\cdot a_{iji^{\prime }j^{\prime }}\leq L-\left(m_{ij}^{1}\cdot l_{ij}+m_{ij}^{2}\cdot w_{ij}\right),\forall i,\forall j,\forall i^{\prime },\forall j^{\prime };$$ (1.9)

$$y_{ij}-y_{i^{\prime }j^{\prime }}+W\cdot b_{iji^{\prime }j^{\prime }}\leq W-\left(m_{ij}^{1}\cdot w_{ij}+m_{ij}^{2}\cdot l_{ij}\right),\forall i,\forall j,\forall i^{\prime },\forall j^{\prime };$$ (1.10)

$$a_{iji^{\prime }j^{\prime }}+a_{i^{\prime }j^{\prime }ij}+b_{iji^{\prime }j^{\prime }}+b_{i^{\prime }j^{\prime }ij}+\left(2-c_{ijt}-c_{i^{\prime }j^{\prime }t}\right)\geq 1,\forall i,\forall j,\forall i^{\prime },\forall j^{\prime };$$ (1.11)

$$c_{ijt},m_{ij}^{1},m_{ij}^{1},a_{iji^{\prime }j^{\prime }},b_{iji^{\prime }j^{\prime }}\in \left\{0,1\right\},\forall i,\forall j,\forall i^{\prime },\forall j^{\prime };$$ (1.12)

Constraint (1.1) represents the maximization of warehouse space utilization, where $\sum_{t}W_{t}\cdot L_{t}$ denotes the size of the warehouse space, and $\sum_{i=1}^{N}\sum_{j=1}^{M_{i}}\sum_{t=1}^{T}c_{ijt}\cdot w_{i}\cdot l_{i}$ represents the warehouse space occupied by various classes of shelving areas.

Constraint (1.2) ensures that at least one type of shelving is placed in each regular area within the warehouse, where *c*_*ijt*_ represents the 0–1 integer variable indicating whether the j-th layout of the i-th type shelves is positioned in the t-th regular area.

Constraint (1.3) ensures that the layout mode of each class of shelves is unique and appears only once in a regular area.

Constraint (1.4) specifies whether the j-th layout configuration of the i-th type shelves is rotated by 90 degrees.

Constraint (1.5) ensures the validity of the x-coordinate for the j-th layout of the i-th type shelves in the t-th area, where *x*_*ij*_ represents the x-coordinate of the bottom-left corner of the j-th layout of the i-th class of shelves, *w*_*ij*_ and *l*_*ij*_ represent the width and length of the j-th layout of the i-th type shelves respectively, *L*_*t*_ is the length of the t-th area, and *X*_*t*_ is the x-coordinate of the t-th area.

Constraint (1.6), along with Eq (1.5), ensures that the x-coordinate *x*_*ij*_ of the j-th layout of the i-th type shelves is within the t-th area.

Constraint (1.7) ensures the validity of the y-coordinate for the j-th layout of the i-th type shelves in the t-th area, where *y*_*ij*_ represents the y-coordinate of the bottom-left corner of the j-th layout of the i-th type shelves.

Constraint (1.8), together with formula (1.7), ensures that the y-coordinate *y*_*ij*_ of the j-th layout of the i-th type shelves is within the t-th area.

Constraint (1.9) ensures that the spatial left-right positions of the j-th layout of the i-th type shelves and the *j*′-th layout of the *i*′-th type shelves do not conflict, and the left-right relationship between them is recorded by the 0–1 variable *a*_*iji*′*j*′_. *L* represents the length of the rectangle covering all areas, where *M* is a sufficiently large positive integer, which could be chosen as 10,000.

Constraint (1.10) ensures that the spatial left-right positions of the j-th layout of the i-th type shelves and the *j*′-th layout of the *i*′-th type shelves do not conflict, and the left-right relationship between them is recorded by the 0–1 variable *b*_*iji*′*j*′_.

Constraint (1.11) ensures that the spatial positional relationship of the j-th layout of the i-th type shelves and the the *j*′-th layout of the *i*′-th type shelves within the largest area conforms to at least one of the four positional orientations: top, bottom, left, or right.

Constraint (1.12) requires that all variables $c_{ijt},m_{ij}^{1},m_{ij}^{1},a_{iji^{\prime }j^{\prime }},b_{iji^{\prime }j^{\prime }}$ be 0–1 variables.

#### 2.2.4. Case study

The warehouse contains three different types of shelves: the first type of shelf is 2 meters wide and 4 meters long, the second type is 6 meters long and 3 meters wide, and the third type is 5 meters long and 3 meters wide. The frequency of these shelves is 5, 6, and 5 respectively.

**Fig 2:**
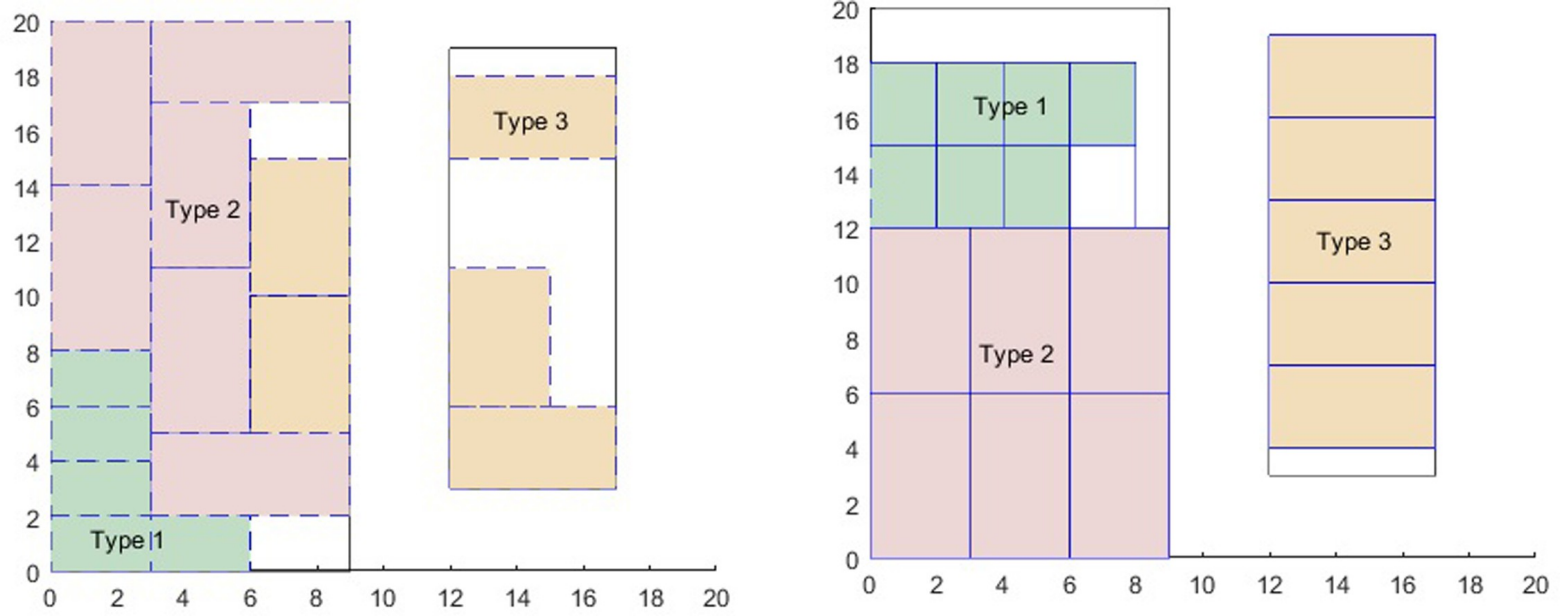
Optimal layout. **(a)** Two-dimensional bin packing problem**; (b) Our new method.**

Based on the existing optimization model for the two-dimensional bin packing problem, we obtained an optimization model with 32 real variables and 512 0–1 variables. After solving the model using Gurobi software, we obtained the optimal layout positions for 16 shelves as shown in Fig 2(A). We found that although the shelves were distributed well in two warehouse areas, the shelves of the third type were scattered in both areas. Obviously, this result does not meet the requirements of the class storage strategy.

By using the clustering algorithm and layout optimization model proposed in this paper, we obtained an optimization model with 32 real variables and 297 0–1 variables. After solving it using Gurobi, we obtained the optimal layout for 16 shelves as shown in Fig 2(B). We found that the three types of shelves were concentrated in two warehouse areas, meeting the requirements of the class storage strategy. Additionally, we found that the number of real variables and 0–1 variables in the new method’s optimization model were reduced by 31.25% and 41.99% respectively.

From Fig 2, it can be seen that based on the clustering algorithm and layout optimization model mentioned above, a class-based storage strategy can be perfectly implemented in the warehouse area. The new method effectively overcomes the challenge in the two-dimensional bin packing problem where similar shelves cannot be grouped together. The new method also overcomes the limitation of existing clustering methods that cannot be converted to non-linear conditions within a quadratic polynomial, which leads to integration issues with the optimization model in the bin packing problem. Additionally, the new method effectively reduces the complexity of the model and improves the computational efficiency.

## 3. Empirical analysis

**Fig 3:**
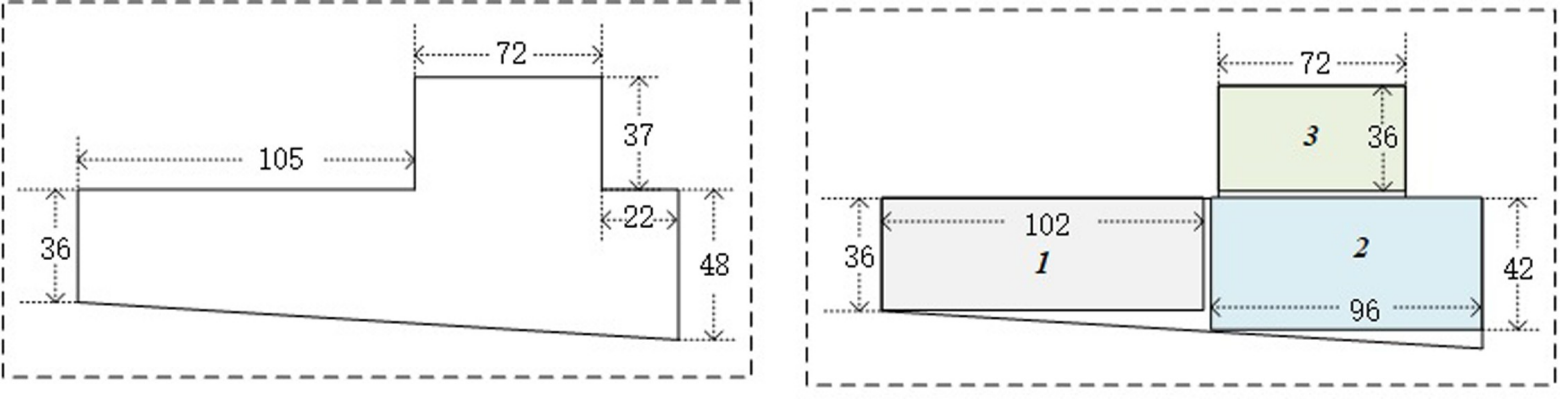
Indoor warehouse area: (a) before division; (b) after division.

The data for this paper are sourced entirely from the Xiangtai Warehouse of State Grid Corporation of China. This terminal warehouse occupies 25,659 square meters, of which the indoor area is 6,570 square meters (Fig 3(A)).

### 3.1. Division of warehouse areas

Following the method described in Transformation of Irregular Storage Areas section, we divided the irregular warehouse area into three regular rectangular areas, with the lengths and widths ${\left\{\left(L_{t},W_{t}\right)\right\}}_{t=1}^{3}$ (m) of the three areas being: (102, 36), (96, 42), and (72, 36), respectively(Fig 3(B)). The bottom-left coordinates ${\left\{\left(X_{t},Y_{t}\right)\right\}}_{t=1}^{3}$ of these areas are (0, 0), (103, 0), and (105, 42). Therefore, we have parameters *L*_0_ as 102m and *W*_0_ as 42m for the algorithm in Clustering Algorithm for Homogeneous Shelves section.

### 3.2. Shelving information

The shelves within the warehouse are categorized into three types: flat storage areas, stacker cranes, and automated storage and retrieval systems (ASRS). Specifically, the flat storage area is divided into four sizes, with the length, width, and frequency parameters ${\left\{\left(l_{i},w_{i},f_{i}\right)\right\}}_{i=1}^{4}$ being (3, 10.6, 24), (6, 4.8, 14), (6, 4.68, 33), and (6, 6, 36). The stacker crane comprises 344 individual shelves each measuring 1.85m in length and 1m in width. The ASRS includes a total of 184 shelves, each measuring 0.9m by 0.9m. The gaps between adjacent shelves, the spacing between rows of shelves, and the width and frequency of aisles are shown in Table 2.

**Table 2:** Information on various types of shelves in the warehouse.

Area	Flat storage area				Stacker crane	ASRS
	Type 1	Type 2	Type 3	Type 4		
*i*	1	2	3	6	4	5
*l*_*i*_	3	6	6	6	1.85	0.9
*w*_*i*_	10.6	4.8	4.68	6	1	0.9
*f*_*i*_	24	14	33	36	344	184
*L*_0_	96	96	96	72	96	96
*W*_0_	42	42	42	36	42	42
*p*_1_	6	6	6	6	6	1
*p*_2_	5.8	5.8	5	6	1	1
*p*_3_	0.1	0.1	0.1	0.1	0.1	0.1
*n*_1_	1	1	1	1	1	2

### 3.3. Empirical results

**Fig 4:**
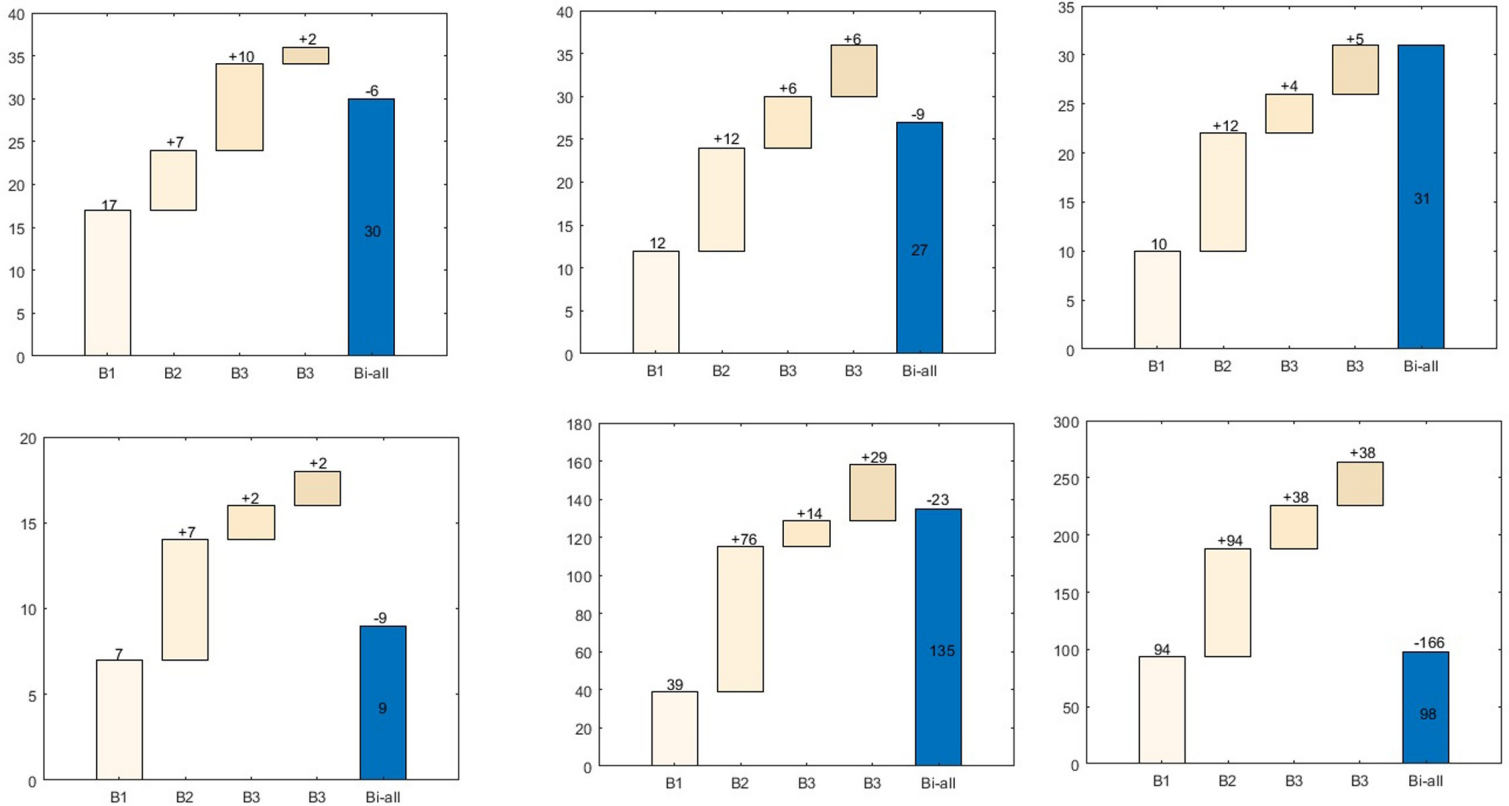
Shelf layout frequency: (a) first type of flat storage area; (b) second type of flat storage area; (c) third type of flat storage area; (d) fourth type of flat storage area; (e) stacker crane; (f) ASRS.

Based on algorithm SAH, we obtained four layout quantities for Type 1 shelves under category (*B*1,*B*2,*B*3,*B*4) as 17, 7, 10, and 2 respectively. Using AAS to eliminate duplicate layouts, we ultimately achieved 30 distinct layouts (Fig 4(A)). Similarly, we completed the layout frequency determination for the other five types of shelves, resulting in frequencies of 27, 31, 9, 135, and 98 respectively (Fig 4(B)–4(F)).

**Fig 5:**
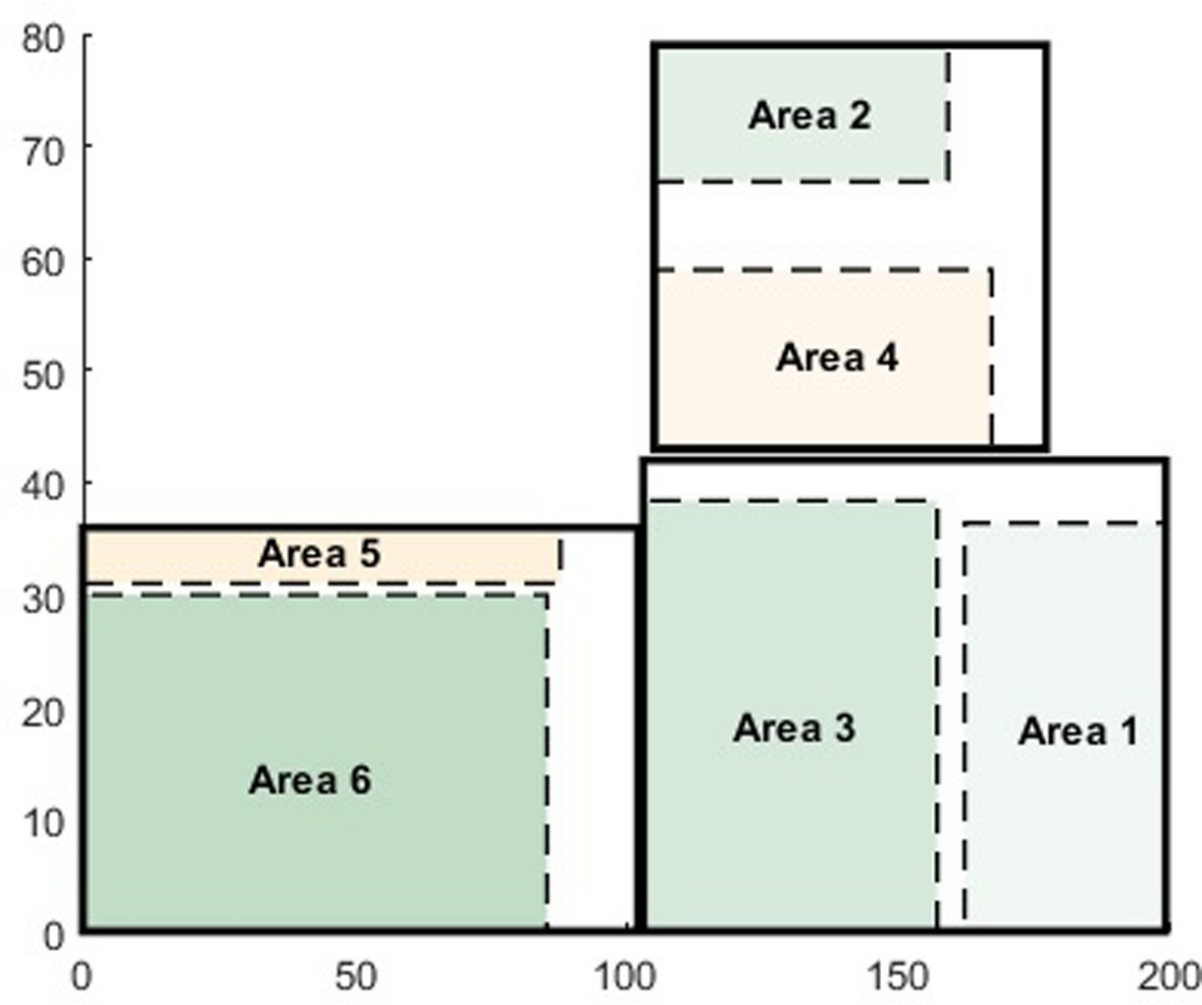
Spatial distribution of various shelving areas within the warehouse.

By substituting the above results into the optimization model (1) in section 2.3, and using the Gurobi software, we obtained the optimal positions for the six types of shelves within the warehouse (Fig 5). In the bottom-left area of the warehouse, there are two types of shelving areas: Area 6, which accommodates ASRS with its bottom-left corner at (0, 31) and dimensions of (87.8, 5); Area 4, which holds the fourth type of flat storage shelves, located at the bottom-left corner (0,0) with dimensions of (85.2, 30).

In the top-right area of the warehouse, the settings are for the stacker crane and the second type of flat storage shelves. The stacker crane is located in Area 5, with the bottom-left corner at (105,43) and dimensions of (62,15.95); Area 2 hosts the second type of flat storage shelves, with the bottom-left corner at (105, 64.2) and dimensions of (54, 12.2).

In the bottom-left corner of the warehouse, there are two shelving regions: Area 3, which hosts the third type of flat storage shelves, its bottom-left corner being at (0, 103) and dimensions of (54,38.4); and Area 1, accommodating the first type of flat storage shelves, with its bottom-left corner at (0,162) and dimensions of (34.4,39).

### 3.4. Model complexity

We solved the layout problem using the multi-bin size packing problem method. After using the Gurobi software, we obtained an optimization model with 1270 real variables and 807500 0–1 variables. However, after running for 26 hours, we did not obtain a result.

In contrast, the optimization model under the new method contains 653 real variables and 140959 0–1 variables, reducing the variables by 48.58% and 82.54% respectively compared to the existing method. Furthermore, the solution time was shortened to 35.97 seconds.

It is evident that the new method not only effectively implements the class storage strategy but also significantly reduces the complexity of the model and effectively shortens the solution time.

## 4. Conclusions

In implementing a class-based storage strategy in warehouse layout, it is necessary to simultaneously determine the classification of materials, the sizes of the different class areas, and their locations. This paper categorizes materials according to the type of shelves and proposes an algorithm that converts the nonlinear clustering problem of homogeneous shelves into a linear problem of selecting regular areas of different shapes and sizes. After transforming the irregular warehouse space into multiple regular rectangular areas and integrating the two-dimensional bin packing problem, a novel optimization model is proposed.

Empirical results based on Xiangtai Warehouse of State Grid Corporation of China show that the proposed solution is effective and feasible, and has general promotional value for the layout optimization problem of irregular warehouse areas under class storage strategy.

## Supporting information

S1 Data (DOCX)
