# Correction: Layout optimization of irregular storage areas under class storage strategy based on clustering and multi-bin size packing problem

**DOI:** 10.1371/journal.pone.0321787

**Published:** 2025-04-02

**Authors:** Wenbin Zhang, Yuejun Jin, Huarong Zhang, Yiming Wang

The second and third authors’ names are spelled incorrectly. The correct names are Yuejun Jin and Huarong Zhang, respectively.

The correct citation is: Zhang W, Jin Y, Zhang H, Wang Y (2024) Layout optimization of irregular storage areas under class storage strategy based on clustering and multi-bin size packing problem. PLoS ONE 19(8): e0307218. https://doi.org/10.1371/journal.pone.0307218.
