# Supplementary material for: Layout optimization of irregular storage areas under class storage strategy based on clustering and multi-bin size packing problem
Source: PLoS One. 2024 Aug 30;19(8):e0307218. doi: 10.1371/journal.pone.0307218 (PMC11364414; doi:10.1371/journal.pone.0307218)
Supplement: S1 Data — (DOCX) [file pone.0307218.s001.docx]

The original dimensions of the warehouse are shown in Figure 1:

**Figure 1. Indoor warehouse area**

The information of the shelves is shown in Table 1:

**Table 1.** **Information on Various Types of Shelves in the Warehouse.**

| **Area** | **Flat storage area** | | | | **Stacker crane** | **ASRS** |
| --- | --- | --- | --- | --- | --- | --- |
|  | **Type 1** | **Type 2** | **Type 3** | **Type 4** |  |  |
| $i$ | 1 | 2 | 3 | 6 | 4 | 5 |
| $l_{i}$ | 3 | 6 | 6 | 6 | 1.85 | 0.9 |
| $w_{i}$ | 10.6 | 4.8 | 4.68 | 6 | 1 | 0.9 |
| $f_{i}$ | 24 | 14 | 33 | 36 | 344 | 184 |
| $L_{0}$ | 96 | 96 | 96 | 72 | 96 | 96 |
| $W_{0}$ | 42 | 42 | 42 | 36 | 42 | 42 |
| $p_{1}$ | 6 | 6 | 6 | 6 | 6 | 1 |
| $p_{2}$ | 5.8 | 5.8 | 5 | 6 | 1 | 1 |
| $p_{3}$ | 0.1 | 0.1 | 0.1 | 0.1 | 0.1 | 0.1 |
| $n_{1}$ | 1 | 1 | 1 | 1 | 1 | 2 |
